# Supplementary material for: MetaRibo-Seq measures translation in microbiomes
Source: Nat Commun. 2020 Jun 29;11:3268. doi: 10.1038/s41467-020-17081-z (PMC7324362; doi:10.1038/s41467-020-17081-z)
Supplement: Supplementary file 10 — Supplementary Data 7 [file 41467_2020_17081_MOESM10_ESM.zip › File2/Confidence_VeryHigh_Taxonomy/84793_out.krona.html]

Javascript must be enabled to view this page.

members
magnitude
magnitudeUnassigned
count
unassigned
taxon
rank

84793\_out

6
3

SRS051031\_contig\_number\_contig-100\_1678.188830SRS053356\_contig\_number\_35136SRS098571\_contig\_number\_65196

2759
2
superkingdom

kingdom
4751
2

2
451864
subkingdom

5204
2
phylum

subphylum
2
5302

155619
2
class

subclass
452333
2

order
2
5338

family
104366
2

genus
2
5320


SRS015190\_contig\_number\_12098SRS019685\_contig\_number\_12161
64638
2
species

superkingdom
1
2

phylum
1
201174

class
1760
1

1
85006
order

1
1268
family

1
1742989
genus

1933880

SRS013687\_contig\_number\_20366
1
species
